# Digital pulse: Social media reaction to South Africa’s National Health Insurance implementation

**DOI:** 10.4102/phcfm.v17i1.4997

**Published:** 2025-09-04

**Authors:** Hlabje C. Masemola, Mutshidzi A. Mulondo, Sphamandla J. Nkambule, Bafana Madida, Raikane J. Seretlo

**Affiliations:** 1 Department of Optometry, Faculty of Health Sciences, University of the Free State, Bloemfontein, South Africa; 2 Division of Public Health, Faculty of Health Sciences, University of the Free State, Bloemfontein, South Africa; 3 Department of Public Health Medicine, Faculty of Health Sciences, University of KwaZulu-Natal, Durban, South Africa; 4 Department of Public Health, School of Health Sciences, Sefako Makgatho Health Sciences University, Pretoria, South Africa

**Keywords:** social media, reactions, implementation, South Africa National Health Insurance, Universal Health Coverage

## Abstract

**Background:**

Social media has become a platform where unheard voices within different communities are shared with government.

**Aim:**

The study explored and described expressed reactions of social media users regarding the implementation of the National Health Insurance (NHI) in South Africa.

**Setting:**

This study was conducted online on existing social media platforms that share current news. These social media platforms included X (formerly known as Twitter), Facebook, Instagram and TikTok.

**Methods:**

This was a qualitative study that applied an explorative-descriptive approach. Using convenience sampling, raw data from screenshots of the first 10 social media users’ reactions from each news media company were collected. The authors collected information verbatim from the screenshots and created two main transcripts with 10 reactions from each of the selected news media accounts. Thematic analysis was used to analyse data.

**Results:**

Eight main themes emerged from the reactions of the users. These include concerns about the public financial and taxation burden, corruption and mismanagement by the government, concerns about the quality of healthcare services, fear of medical staff exodus, issues of equity and access to healthcare, government’s political motives and electioneering, user’s preference for improving current public healthcare system and doubts about NHI implementation feasibility.

**Conclusion:**

The findings emphasise the need for government officials to include the community before introducing, signing and implementing different bills.

**Contribution:**

Through highlighting the public’s sentiments on challenges, readiness and feasibility of implementing the NHI, policymakers will be encouraged to ensure adequate health communication and community participation.

## Introduction

Strengthening the health system in consideration of global health initiatives is paramount in a bid to enhancing the efficiency, effectiveness and equity of healthcare systems worldwide. Achieving Universal Health Coverage (UHC) requires strong health systems according to the World Health Organization (WHO).^1^ Within South Africa’s context, the National Health Insurance (NHI) fund represents a significant policy that endeavours to fundamentally transform the nation’s health system to realise UHC.^2^

The existence of state versus privately funded healthcare has been a key characteristic of South Africa’s divided and fragmented health system, stemming predominantly from significant historical disparities. Within the bifurcated system, 83.7% of the total population is served by free state-funded services riddled with underfunding, resource unavailability and infrastructure challenges – perpetuating continual disparities in health outcomes.^3^ Conversely, the well-resourced private sector remains accessible primarily to households that can afford medical schemes, serving 16.3% of the total population.^4^ Through the creation of a more equitable health system, the NHI represents a policy intervention that seeks to bridge this gap by guaranteeing all South Africans, irrespective of socioeconomic level, access to comprehensive and quality healthcare care past primary health care.^5^

In aligning with the WHO UHC guidelines, the South African government proposed and passed the NHI Bill in 2023, which was finally signed into law in May 2024. Though the concept of a ‘National Health Insurance Bill’ spans from 1994 in its conceptualisation – with key landmark events (i.e. Green Paper in 2011 and White Paper in 2015) – it has repeatedly elicited criticism from a plethora of stakeholders. Critics, which include healthcare professionals, the private sector, civil society, academia and policy analysts, have raised concerns about the government’s ability to execute the NHI Bill in an efficient and financially sustainable manner.^6^ Such public opinion plays a crucial role in bill reformations as a key mediator in the discourse.^7^ In this digital age, social media has become a potent tool for public engagement, knowledge sharing, health communication and public perception influence.

**FIGURE 1:**
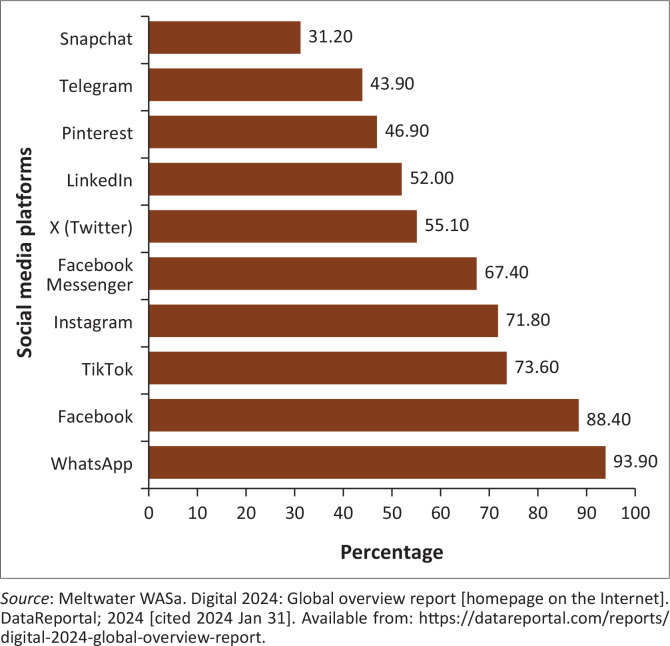
Most used social media platforms in South Africa by internet users aged 16–64 years, based on monthly usage (amended from GWI).

Bhanye et al. describe social media as a ‘valuable repository of information’, a ‘hyperspace’, a ‘new world’ and a ‘form of currency’.^8^ The use of social media in South Africa has been integrated into the public’s way of living.^9^ The Digital Global Overview Report (2024) indicates that South Africa has 45.34 million Internet users with 42.8% of them using social media to find information (83.6%), stay in touch with friends and family (72.5%), keep up to date with news and events (66.2%) and research health issues and healthcare products (53.2%), as detailed in Figure 1.^10^ Social media in South Africa has demonstrated the possibility of fostering not only public discussion but also organise movements to mobilise opinions on social and political issues in a bid to stimulate civic conversations and hold government accountable.^11^

Analytics into social media offer valuable insight for researchers to identify and understand public opinions, perceptions, concerns and support for policies in a cost-effective manner.^12^ Unlike traditional paper-based surveys that have inherent limitations such as sample size, closed questions and limited geographic and temporal data, the application of social media analysis provides real-time sentiments and attitudes with enhanced spatiotemporal detail.^13^ Computational studies, such as sentiment analysis, allow researchers to take opinions, sentiments and emotions in their text form expressed on media platforms and translate their polarity (positive, negative and neutral).^14^ Furthermore, through Natural Language Processing (NLP), techniques and machine learning algorithms can extract emotional responses (i.e. angry, sad and happy) or state of mind (i.e. interested vs. disinterested) from unstructured public opinion data into consistent thematic representations within a subject matter.^15^

Limited studies within South Africa have used sentiment analysis to examine public opinion towards NHI in South Africa from a temporal point. Struweg identified the South African government (Ministry of Health), the media and its citizens as key drivers in the NHI discourse shortly after its announcement for discussion in parliament in 2019.^16^ To the best of our knowledge, this is the first study assessing sentiments towards the implementation of South Africa’s NHI. This study contributes to the SDGs by providing empirical data on public sentiment regarding the NHI, thereby supporting SDG 3, which aims to achieve UHC. By analysing social media trends, this research will also contribute to SDG 16, which promotes inclusive and participatory decision-making processes. Furthermore, the findings can aid in enhancing the implementation of health policies, thus supporting SDG 10, which focuses on reducing inequality within and among countries.

## Research methods and design

### Study design

This was an explorative-descriptive qualitative study, as the authors aimed to understand the expressed reactions of social media users regarding the implementation of the NHI in South Africa.

### Study settings

This study relied on secondary data, as there was no physical setting. The study was conducted on various social media platforms, including X (formerly known as Twitter), Facebook, Instagram and TikTok. Twenty-seven South African media rooms were utilised to access the secondary data based on the availability of the post regarding NHI.

### Population and sampling strategy

The population of this study comprised all individuals with access to social media, irrespective of age, as age is not verifiable. Convenience sampling was used based on what a news media company posted, and the authors selected the first 10 reactions from each news media company. All South African media rooms that posted matters about NHI implementation in May 2024 were included. All other non-South African media rooms talking about NHI were excluded, as the reactions and comments were mainly focused on the views of South African media rooms. All South African media rooms that neither posted nor had any comments about the NHI implementation or signing were also excluded from the study. Lastly, all the information from the South African media room in the form of print was excluded as the study was mainly focused on online content.

### Data collection

Authors reviewed different social media platforms. These social media platforms included X (formerly known as Twitter), Facebook, Instagram and TikTok. Within these platforms, 27 South African media houses were searched and included in this study based on the post each media house posted. The authors backdated the search from 13 May 2024 to 15 May 2024, which was mainly the date of the announcement for NHI signing by the President of the Republic of South Africa. On finding the post, the authors systematically selected the first 10 reactions and comments from social media users regarding the announcement. Some of the questions posted by the South African media rooms were:

‘President … has given a brief account on how government plans to find the implementation of NHI; president Cyril Ramaphosa has officially signed the contentious NHI bill into law despite the many objections it faces; the South African president is expected to sign NHI bill into law this week; president signs the NHI into law at the union building in Tshwane; and where will money come from, on Wednesday, the president “so and so” will sign the NHI bill into law.’

Data collection was stopped when there were no available posts regarding NHI from the selected South African media rooms.

### Data analysis

Data were analysed using thematic content analysis. The authors viewed all the raw data, which were social media users’ reactions and comments as ‘transcripts’. Raw data included screenshots of the different users’ reactions. The authors collected the information verbatim from the screenshots and created two main transcripts with the first 10 reactions from each news media account. All transcripts were imported into the NVivo 14 software and continued with coding and creating themes. Authors created codes based on the similarities. Lastly, the authors identified and interpreted meaningful themes, which were presented as the qualitative findings (refer to Table 1).

**TABLE 1:** A summary of emerged themes.

Themes	
1.	Financial concerns and tax burden
2.	Corruption and mismanagement concerns
3.	Quality of healthcare services
4.	Fear of medical staff exodus
5.	Equity and access to healthcare
6.	Political motives and electioneering
7.	Preference for improving the current public healthcare system
8.	Doubts about NHI implementation feasibility

NHI, National Health Insurance.

### Trustworthiness

All the principles of trustworthiness were followed and applied. For transferability, authors collected enough data from different social media platforms to reflect the broader population of social media users in South Africa. Additionally, the authors outlined all the processes followed in detail so that other researchers can apply them in their studies. A team of five authors worked together during data collection to ensure credibility, quality and rich data. Conformability was achieved by following the stipulated process of data analysis logically. Lastly, to ensure dependability, the authors used an independent coder to serve as a reviewer after data collection and analysis.

### Ethical considerations

Ethical clearance to conduct this study was obtained from the University of the Free State, Health Sciences Research Ethics Committee (No. UFS-HSD2024/1503/2708). No consent form was required as the study applied secondary data from the various social media platforms. To ensure confidentiality, social media users’ real names or handles were not used. To ensure confidentiality and anonymity, the authors used pseudo-names such as X (947), which represent a social media platform and the news media company where users reacted, along with the number of users, thereby upholding the *Protection of Personal Information Act* (POPIA).

## Results

Eight main themes emerged during data analysis. Table 1 illustrates a summary of themes that became evident.

### Theme 1: Financial concerns and tax burden

Many social media users have serious concerns about the NHI’s ability to remain financially viable. Many believe that raising taxes is the only option to finance such a huge financing system for health because the government lacks the resources to carry it out successfully. With numerous references to growing living expenses, inflation and the unemployment rate, users express concern about the growing financial strain on the middle class. It is believed that funding the NHI will make already severe economic challenges worse and that the government is overly dependent on tax income:

‘Building with which money, your government only generates income through taxes yet you want to come up with something that requires money but don’t know where to get it.’ (P3, X, @Dailymaverick)

‘On top of higher interest rates, rising cost of living, high fuel prices, high inflation, low disposable income, ect.’ (P7, X, @Mail&Guardian)

‘It’s not free I’m being taxed for it.’ (P10, Facebook, @News24)

### Theme 2: Corruption and mismanagement concerns

One of the most common issues raised by social media users was corruption by government officials. Many felt that, similar to other state-owned entities’ failures (e.g. Eskom, South African Airways [SAA]), the NHI fund would turn into another ‘cash cow’ for government officials. It is believed that even with the best of intentions, a policy will be unsuccessful because of inadequate administration, bad governance and a lack of accountability. The government’s capacity to manage such a large responsibility without significant financial leaks is also questioned:

‘This bill will inevitably lead to corruption, looting of new NHI piggy bank …’ (P4, X, @enca)

‘They neglected and looted public hospitals! It’s gonna be a mess.’ (P3, X, @News24)

‘Maladministration and looting in every level of government. All the NHI is, is state capture on an unprecedented level. If you thought the abuse of COVID funds was horrendous, what will pale into insignificance under the NHI.’ FB (Moneyweb)

### Theme 3: Quality of healthcare services

The discourse was dominated by users’ concerns about the current status of public healthcare facilities, with many saying that government-run clinics and hospitals are already experiencing a crisis as a result of staff shortages, long waiting times and lack of funding. With the public system currently in turmoil, there is uncertainty on whether the government can provide good-quality healthcare under the NHI. Many people are concerned that healthcare standard would drop and that this might compromise the private healthcare system:

‘They can’t even manage the hospital they’re currently responsible for.’ (P1, X, @enca)

‘Public hospitals are with excessively long queues where it takes months to get anything done.’ (P5, X, @BusinessTechSA)

‘Currently, the government already cannot sustain the state of hospitals.’ (P2, Facebook, @News24)

### Theme 4: Fear of medical staff exodus

A large number of users fear that because of possible salary cuts, greater workloads and inadequate incentives, NHI would cause a mass exodus of South African healthcare professionals. Some predict that the nation’s healthcare system will have trouble keeping qualified professionals, which will cause healthcare services to continue to deteriorate:

‘Get ready for mass emigration of doctors and nurses.’ (P9, X, @BusinessTechSA)

‘Doctors who are going overseas, where exactly are they going? Do people know how good doctors are paid in our public hospitals?’ (P6, Instagram, @947Station)

‘No specialist healthcare professionals will stick around for this.’ (P1, Facebook, @News24)

### Theme 5: Equity and access to healthcare

Some social media users are in support of NHI, pointing out that it will give everyone in South Africa, especially the underprivileged and those without medical aid, equal access to healthcare. They believe that the NHI can lessen the disparities that now exist between the public and private healthcare sectors and that healthcare is an essential human right. There are, however, disagreements that maintain that those who currently receive private healthcare benefits may suffer if healthcare resources are redistributed:

‘It means equal access to advanced health care.’ (P8, X, @Mail&Guardian)

‘Welcome NHI. Welcome equal access to healthcare, to all welcome.’ (P9, X, @News24)

‘The outrage we see is about disadvantaged persons receiving equal health to the rich. There’s no sound basis against NHI.’ (P2, X, @BusinessTechSA)

### Theme 6: Political motives and electioneering

The majority of the social media users perceived the signing of the NHI bill into law as a method of the South African ruling party to win votes as a campaigning tool during Election Day. Additionally, some social media users showed some disappointed reactions that politicians use real life-changing experiences just to capture and distract voters into believing the ruling party cared for them:

‘The things they do now for votes, this is sad and the final straw.’ (P2, Instagram, @NBnewswrap)

“He is doing it to buy poor people’s votes …’ (P4, Instagram, @Brieflynews)

‘The public signing of the National Health Insurance Bill into an Act was just an electioneering puppet show by President Cyril Ramaphosa of the ANC hoping people to believe that they can now go to any private hospitals without medical aid & thus get some votes after its signing.’ (P1, X, @EyewitnessNews)

‘This is for votes, it will take years to actually come into effect, people don’t know they will be paying for it and a lot will get turned away as they don’t have funds, yet hopefully before the voting people, we see this as a huge scam.’ (P8, X, @enca)

### Theme 7: Preference for improving the current public healthcare system

There were various suggestions from the comments of the social media users that the government should instead focus on refining and strengthening the existing healthcare system rather than introducing something new that might fail as the current healthcare system has. Most of the social media users felt that the South African government could use the existing resources to enhance existing hospitals, clinics and staff levels before introducing the NHI:

‘The current public health system is a mess, so I don’t see how this is going to be any different. Sounds good to a certain extent but I doubt it’ll be that good in reality.’ (P4, Facebook, South African Government)

‘Who is going to pay for those bills? The government should have fixed the public hospital. U can’t have a wife and buy takeaway every time. So, there will be money that will be claimed by the government continuously. Government is renting health care now instead of improving their health care system.’ (P10, Facebook, SABC News)

‘Why not fix the public hospital to be a level of private hospitals before signing things that are going to disrupt the country even more, this guy flies to Russia when he’s sick he will not utilise this NHI thing, we are already paying a lot of money for tax, and you still want to milk us more.’ (P4, Facebook, @enca)

‘Why can’t they just fix the public health care system on their own and have them compete against the private sector?’ (P3, Instagram, @NBnewswrap)

### Theme 8: Doubts about National Health Insurance implementation feasibility

Almost all the social media users in this study had doubts about NHI implementation in South Africa, some related to how corruption has messed up many great bills and emphasised that if South Africa could not provide basic services like employment to its doctors and nurses, then the NHI is a dream. Lastly, some social media users were sceptical about the government’s ability to deliver on its promises, and some compared it with other failed existing governmental entities and initiatives:

‘Currently, the government already cannot sustain the state of hospitals. The state hospitals are severely lacking equipment, staff, and necessities. Now, they are going to pump more money into a system similar to one that already doesn’t work??!!! Not to mention that there are doctors who cannot get jobs because of lack of funding. This makes no sense. Is it just going to mean that more of us get even worse medical treatment??’ (P2, Instagram, @News24)

‘This NHI will collapse like Eskom. How can you raise the tax by working class (employed) if our jobless rate is one of the highest in the world!! First, fix the number of people who don’t have a job before you try this NHI scheme. If NHI doesn’t work abroad what makes you think it will work in SA?’ (P5, Instagram, @News24)

‘We have a public health system that is mismanaged by incompetents, add to that the inflated cost of the BEE tenderpreneur system and all the border jumpers coming in and accessing our public health system at the tax-payers expense. Thus, is unsustainable.’ (P7, Facebook, Moneyweb)

‘It will never work in SA there is too much corruption and also not enough people contributing to the system and the people who are already paying so highly at the moment are going to be hit harder, not fair for people who are already struggling to make ends meet …’ (P9, Facebook, National Department of Health)

## Discussion

Implementing South Africa’s NHI has ignited a complex and often contentious debate on social media, reflecting deep-seated, polarised public discourses.^17^ This study identified eight primary emerging themes reflecting context-specific concerns, challenges and support for the NHI, including financial viability, corruption fears, healthcare quality, medical staff migration, equity in access, political motivations, the preference for improving the current system, and doubts about the feasibility and potential benefits thereof.

These findings align with global discussions on universal healthcare implementation, where financial sustainability, governance and quality assurance remain major concerns.^18,19^ In this section, the results will be discussed under each theme to contextualise the findings within the broader literature on health system reforms and UHC.

### Financial concerns and tax burden

The study found that the most prominent theme emerging from social media commentary is the financial concerns and tax burden the NHI may place on taxpayers, underscoring the public’s anxiety about the NHI’s sustainability. This aligns with findings in other contexts where healthcare reforms are met with skepticism regarding funding sources and economic impact.^20,21,22,23,24^

Many users expressed skepticism about the government’s ability to fund the system without disproportionately increasing taxes – a concern echoed in the broader literature on UHC.^24,25^ The fear of increased taxes, particularly among the middle class, is consistent with research showing that tax-funded healthcare systems can face significant public resistance because of perceived economic burdens.^26,27,28^

The social media commentary in South Africa demonstrates a pervasive distrust in the government’s fiscal management, a sentiment exacerbated by the current economic climate in South Africa, characterised by high unemployment and inflation.^29,30,31^ Furthermore, apprehensions concerning corruption and administrative deficiencies additionally compound these financial concerns.

Contrary to this public sentiment, the South African healthcare leadership expresses confidence in its political leadership, enduring commitment, and proactive, adaptable strategies to facilitate a shift towards the NHI, thereby promoting UHC.^17,32^ This discrepancy highlights a fundamental disparity in perspectives between those in leadership and governance roles and deep-seated concerns and expectations among the populace, specifically concerning the level and efficacy of stakeholder engagement.

### Corruption, mismanagement concerns and governance issues

Corruption is a well-documented obstacle to effective policy implementation and public sector reform in developing countries, and this pervasive concern significantly undermines public trust in South Africa’s proposed NHI. Similar challenges have been observed in countries like Nigeria and Kenya, where NHI schemes faced allegations of fund mismanagement, reducing public trust and limiting effective implementation.^33,34,35,36,37^

While governance scandals have plagued South African public institutions, the skepticism surrounding the government’s capacity to manage the NHI fund efficiently is particularly salient. This skepticism mirrors broader critiques of large-scale government programmes, notably the failures of state-owned enterprises like Eskom and SAA.^38,39^ The social media discourse reveals a deep-seated fear that the NHI will become another vehicle for state capture and financial malfeasance, echoing observations made by Transparency International and other anti-corruption organisations.^36,40,41,42,43,44^

### Quality of healthcare services and fear of medical staff exodus

Public concerns regarding the quality of healthcare services under the proposed NHI were also prevalent. Many social media users emphasised the existing deficiencies within the public healthcare system, arguing that the government should prioritise improving the current infrastructure before implementing an ambitious national insurance plan. This skepticism about the NHI’s ability to enhance healthcare quality is deeply rooted in the current state of public healthcare facilities.

The current state of public healthcare facilities is characterised by widespread dissatisfaction with long wait times, staff shortages and inadequate resources in state-owned hospitals, reflecting findings from numerous studies on healthcare access and quality in South Africa. For instance, reports by South Africa’s Department of Health and academic research by the Global Burden of Disease 2019 Study and the Health Systems Trust’s South African Health Review 2023 (SAHR) consistently document these challenges.^45,46,47^ This sentiment aligns with research from Thailand’s universal healthcare system, which faced initial challenges in service delivery because of inadequate facilities and workforce shortages.^19,48^ Critically, the fear that the NHI will lead to a decline in healthcare standards, even in the private sector, reflects a lack of confidence in the government’s ability to integrate and manage a unified healthcare system effectively.

This fear extends to the potential exodus of medical professionals, a critical issue that could further undermine the NHI’s success. Many fear salary reductions, increased workload and poor working conditions under NHI. Similar challenges have been reported in Ghana’s NHI Scheme, where healthcare professionals migrated to better-paying private and international opportunities, undermining service delivery in public hospitals.^49,50,51^ This aligns with healthcare worker migration and retention research, which shows that inadequate compensation, poor working conditions and a lack of professional development opportunities can drive healthcare professionals to seek employment elsewhere.^52,53^

Overall, the social media commentary reflects a concern that the NHI’s implementation will exacerbate existing challenges in retaining skilled healthcare staff and the current state of public healthcare facilities, thus potentially leading to further deterioration of healthcare services. If not adequately addressed, this could lead to declining healthcare standards and exacerbate workforce shortages in South Africa.

### Equity in healthcare access versus political motives

While concerns about potential decline in healthcare quality and the exodus of healthcare professionals dominated social media discussions, some users supported the NHI, emphasising its potential to equalise access and reduce disparities between public and private healthcare.

Universal healthcare, often framed as a fundamental human right, has demonstrated its ability to reduce health inequalities in countries like Thailand and Costa Rica (Knaul et al., 2012).^54,55,56^ However, opposition to the NHI revealed a fear that resource redistribution might compromise care quality for those accustomed to private services. This tension between equitable access and maintaining existing private healthcare standards emerged as a central challenge. Simultaneously, strong support for the NHI’s goal of universal access to quality healthcare, aligning with established principles of UHC,^57^ was also evident. Ultimately, the debate underscored the necessity of balancing competing interests, ensuring quality and workforce retention while expanding access to underserved populations for successful NHI implementation.

Adding to the scepticism surrounding the NHI, many users perceived its implementation as an electioneering tactic, questioning the timing of the bill’s enactment. This perception, prevalent in other countries where healthcare reforms are introduced to gain political mileage,^58,59^ highlights the politicisation of healthcare reform in South Africa. Research on the political economy of healthcare reinforces this, demonstrating how political considerations and electoral cycles often influence healthcare policies.^60,61,62^ The social media commentary reflected a widespread cynicism about the government’s intentions, with many viewing the NHI as a vote-buying strategy rather than a genuine effort to improve healthcare. This scepticism could further weaken public trust in the policy’s intentions if left unaddressed.

### Doubts about the feasibility and alternative policy recommendations

Widespread doubts regarding the NHI’s feasibility centred on the government’s perceived incapacity to manage such a large-scale reform. Many users advocated prioritising the strengthening of the existing healthcare system, a strategy aligned with international best practices. A gradual enhancement of healthcare infrastructure and governance often precedes successful universal healthcare reforms.^30,56,63^ This suggests that a phased approach, ensuring system readiness before comprehensive rollout, may be more prudent for South Africa. The social media commentary revealed a strong sentiment that addressing current systemic weaknesses should precede any ambitious new national healthcare initiative.

This advocacy for improving the existing public healthcare system reflects a pragmatic approach, emphasising addressing current challenges before embarking on significant reform. Such a perspective is supported by research on health system strengthening, highlighting the importance of leveraging existing infrastructure and resources.^47,56^ The prevalent lack of confidence in the government’s delivery capacity, fuelled by perceived failures in other state-led initiatives and ongoing public service delivery issues, further underscores the need for a phased NHI implementation. This phased approach was detailed by South Africa’s President during the State of the Nation Address in 2025, which includes, among other recommendations, the establishment of a Ministerial Advisory Committee on health technologies and healthcare benefits.^64^ Building trust through tangible improvements in current healthcare services is crucial to mitigating scepticism and ensuring public buy-in for future reforms.

### Study strengths and limitations

This study offers valuable insights into public perceptions of the NHI by analysing social media discourse, a platform of growing influence for policy debate. Thus, capturing real-time public sentiments contributes to the expanding body of research on digital health policy discussions.

However, while social media provides a dynamic window into public opinion, several limitations necessitate a cautious interpretation of the findings. Specifically, this study’s reliance on South African online media platforms excludes print media and international perspectives, potentially overlooking crucial contextual elements.

Furthermore, the variability of NHI-related reactions across online platforms suggests that certain public viewpoints may have been missed. Beyond these limitations in scope, the inherent nature of social media data introduces further considerations. Social media users tend to be younger and more digitally literate, potentially skewing the sample away from marginalised or less digitally connected communities. Additionally, while effective, the thematic analysis employed carries the potential for subjective interpretation.

### Future research directions

To address these limitations and achieve a more comprehensive understanding of public sentiment, future research could adopt a mixed-methods approach, integrating traditional media analysis and survey-based data to ensure a more representative and robust assessment. Furthermore, future research should expand its scope to explore the perspectives of diverse stakeholders, including healthcare professionals, patients and policymakers. Employing both qualitative and quantitative methods will be crucial in capturing the nuances of their experiences and opinions.

Beyond simply assessing sentiment, it is imperative to examine the real-world impact of the NHI’s implementation. Studies should investigate the long-term effects on healthcare access, quality and equity, providing empirical evidence of the policy’s effectiveness. Finally, research should delve into the critical role of public engagement and communication in fostering trust and support for healthcare reform. Understanding how to effectively communicate policy changes and engage with the public is essential for successful implementation and long-term sustainability.

### Implications for policy and practice

The findings have significant implications for policymakers and healthcare stakeholders:

- **Financial transparency and sustainability:** Addressing public concerns over financial feasibility by clearly outlining how NHI will be funded without overburdening taxpayers.
- **Combating corruption:** Implementing stringent governance mechanisms to prevent financial mismanagement and corruption within the NHI fund.
- **Healthcare infrastructure and workforce retention:** Investing in public hospitals, retaining skilled professionals and ensuring working conditions are favourable before implementing NHI.
- **Public engagement and education:** Engaging with the public to clarify misconceptions, address concerns and build trust in the policy.
- **Phased implementation:** Learning from international examples, a stepwise approach may be more effective in rolling out NHI without overwhelming existing healthcare structures.

## Conclusion

The social media discourse surrounding the NHI reveals a complex interplay of deep-seated concerns, expectations and political dynamics. Key concerns centre on financial sustainability, governance, healthcare quality and workforce retention. While the policy garners support for its potential to equalise healthcare access, significant scepticism persists regarding its feasibility and perceived political motivations. Addressing these challenges will require a concerted effort to build trust, enhance transparency and demonstrate tangible improvements in healthcare services.

Policymakers must prioritise strengthening the existing public healthcare system, addressing corruption and ensuring that the NHI’s implementation is guided by evidence-based decision-making and meaningful public engagement. To ensure successful implementation, transparent governance, robust financial sustainability measures and significant infrastructure improvements are essential. Future studies should incorporate diverse media sources and more comprehensive public opinion analysis, including the perspectives of diverse stakeholders, to enrich the discourse surrounding healthcare reform and provide a more nuanced understanding of public sentiment.
